# Should psychiatrists ‘Google’ their patients?

**DOI:** 10.1192/pb.bp.114.047555

**Published:** 2015-12

**Authors:** G. Alice Ashby, Aileen O'Brien, Deborah Bowman, Carwyn Hooper, Toby Stevens, Esther Lousada

**Affiliations:** 1 South West London and St George's Mental Health Trust; 2 Institute of Medical and Biomedical Education, St George's, University of London; 3 St George's, University of London

## Abstract

Since its beginnings in the 1980s the internet has come to shape our everyday lives, but doctors still seem rather afraid of it. This anxiety may be explained by the fact that researchers and regulatory bodies focus less on the way that the internet can be used to enhance clinical work and more on the potential and perceived risks that this technology poses in terms of boundary violations and accidental breaches of confidentiality. Some aspects of the internet's impact on medicine have been better researched than others, for example, whether email communication, social media and teleconferencing psychotherapy could be used to improve the delivery of care. However, few authors have considered the specific issue of searching online for information about patients and much of the guidance published by regulatory organisations eludes this issue. In this article we provide clinical examples where the question ‘should I Google the patient?’ may arise and present questions for future research.

## Background

The internet is a part of everyday life and access to it is growing at a startling rate. According to the United Nations' specialised agency for information and communication technologies (the International Telecommunication Union), by the end of 2014, 3 billion people, approximately 43% of the world's population, will have access to the internet, compared with 60 million people in 2000. The number of mobile-broadband subscriptions will reach 2.3 billion globally by the end of 2014, almost five times as many as in 2008.^1^ Social media has also weaved its way into people's lives over the past decade. There were 802 million daily active users and 1.28 billion monthly active users of the social media site Facebook in March 2014, and over 80% of the daily users of Facebook are based outside the USA and Canada.^2^ According to the Office for National Statistics, the UK has one of the highest rates of social media use in Europe, with almost half of all adults (48%) using social networking sites such as Facebook and Twitter.^3^

Some aspects of the internet's impact on medicine have been better investigated than others. Various authors have looked at whether email could be used as an acceptable form of communication between doctor and patient.^4,5^ Psychiatrists have also looked into the possibility of using video teleconferencing for psychotherapy.^6^ The use (and misuse) of social media by doctors, in terms of professionalism and boundary violations, has been studied.^7,8^ This has led to a plethora of professional regulatory bodies and other medical organisations publishing guidelines to help doctors navigate the internet in general and social media in particular.^9-13^ The published guidance is reasonably thorough: guidelines concentrate on the advice given to professionals about their use of social media sites, and their profile on them. Professionals are advised against forming friendships with patients online. However, professional bodies are often behind the curve in doing this. For example, the UK's General Medical Council guidelines were first published on the 25 March 2013,^9^ a full 9 years after Facebook was founded.^14^

The issue of searching online for patient information has caught the imagination of doctors and ethicists writing in the blogosphere.^15-17^ Many raise concerns about the potential ethical ‘slippery slope’ of Googling your patient, but raise examples where it may be justifiable. However, there has been little discussion in formal medical ethics and law literature about this issue. There is also a paucity of official guidance to help doctors navigate their way through this particular online minefield. For example, the British Medical Association's guidance on social media does not refer to this issue at all and the Royal College of Psychiatrists in the UK has not yet issued any guidance in relation to this kind of online activity. Where professional guidance does touch upon the issue of ‘online searching’ it focuses on the problems that can arise when patients ‘Google’ their doctors rather than the other way around. We are not aware of any legal cases in this particular area either. In sum, there is an apparent regulatory lacuna in terms of whether and if so, how, doctors should use the internet to dig around in their patients' digital backyards.

The lack of research and guidance in this area is increasingly problematic because a great deal of information about patients is now available at the click of a search box button. Many of us are documenting our lives online far more than might have ever been expected 20 years ago and doctors and patients can now find out a huge amount about each other online with relatively little effort.^18^ In addition many patients post personal information online that could potentially harm them or have an impact on clinical assessments by their healthcare professionals and a growing number of doctors admit to using the internet to search for general clinical information.^19^

Clinton *et al* provide the only comprehensive review of the literature on the ethical difficulties surrounding searching online for information about patients, something they term ‘patient-targeted Googling’ (PTG).^20^ They provide a list of questions for psychiatrists to consider before deciding whether to use PTG, shown in Box 1.^20^ They argue that PTG can be an acceptable clinical tool but warn against ‘unbridled PTG simply because online information is legally available in the public domain’. They describe possible conflicts, such as using ‘Google Earth’ to look at photographs of a patient's large house, when the person has not paid for psychotherapy (the authors are based in the USA), and compare this with driving past the patient's house, which would be considered by many to be a boundary violation.^20^ This raises questions about whether such media reconstitute the meaning of boundary violation: is looking at ‘Google Earth’ as intrusive as going to look at someone's house in person? The internet, whether we like it or not, has caused pre-existing boundaries to blur.

In this paper we will look at the potential benefits and harms related to internet searching being used as a clinical investigative tool and propose some questions for future research. In this article we use the word ‘Google’ as a verb intentionally, as it has become part of our everyday English language, meaning ‘to use an internet search engine to find information’.

## Checking conflicting and falsified information

Internet searching for information about patients may mean finding things you did not expect, that the patient had not shared or had even lied about. Should we use the internet to investigate factitious disorder or malingering?

Volpe *et al* discuss a case involving a 26-year-old patient who had requested a prophylactic bilateral mastectomy with reconstruction because of an extensive family history of cancer, where there was suspicion among the clinical team that some of the history was fabricated.^21^ In the paper Volpe, Blackall and Green argue that ‘uninvited patient Googling’ is bad practice for three reasons. First, it bypasses the personal relationship and makes it too easy to terminate a relationship with a patient, and to avoid discussion of personal topics. Second, it erodes provider-patient trust. Third, it represents an invasion of privacy. They also make the point that it is unclear why a healthcare professional would not just ask the individual in person if they had any concerns about them. In the same paper, George, Baker and Kaufmann argue for the opposite position.^21^ They note that it would be ‘irresponsible not to exhaust all resources in learning about a patient with such troubling red flags’. They argue that the finding of a factitious disorder via the patient's two Facebook pages ‘saved a team of professionals from aiding and abetting a fraudulent, deceptive and self-injurious scheme’, stopping them from breaking their oath to first ‘do no harm’.^21^

Clinton *et al* ask how the discovery of important information found online would then be broached with the patient and how this information should be documented in the medical notes.^20^ Interestingly, no author we could find in a literature search had considered whether an online search could be performed with the patient's informed consent and, perhaps, in the patient's presence. In the Volpe *et al* case, for example, the surgical and genetics teams used an internet search in what they believed to be the patient's best interests, without telling her beforehand. What is also not explained is how the patient was told she would not be having surgery and what reasons the patient was given for this decision.

## Uncovering dangerous lifestyle choices

Doctors could Google their patients in order to investigate concordance with advice about treatment and lifestyle changes, including advice about not driving or misusing various drugs. Psychiatrists, in particular, might be interested in discovering whether a patient with psychosis is drinking alcohol heavily or using other substances, which might cause or exacerbate psychotic symptoms.

Farnan *et al*'s main concern is that ‘digitally tracking the personal behaviours of patients, such as determining whether they have indeed quit smoking or are maintaining a healthy diet, may threaten the trust needed for a strong patient-physician relationship’.^22^ The violation of trust might occur because patients assume that doctors do not perform such searches (i.e. the violation relates to a real, or perceived, deceit) or because they feel that such activity violates important boundaries. Gabbard *et al* note that the boundary violation may be the nub of the problem.^23^ It is hard to imagine how the doctors in the Volpe *et al* case, described above, approached their patient with the information found on her Facebook accounts. If the information was related to her – as it presumably was – it is hard to see how this could have led to a positive, therapeutic, outcome. Indeed, the patient may well have felt betrayed by the team caring for her.

Of course, if a doctor can find out about such things as alcohol and drug misuse by searching in the ‘online public domain’, the same holds true for other people. Accessing information on an internet search engine or social media site would be much easier for a patient's future employer than accessing a person's medical records without their consent. In an era of recovery-oriented medicine, including supporting people to return to work, perhaps we should be proactively and openly discussing online presence, for example as part of the employment support provided by a community psychiatry team?

## Mistaken identity

Another problem may arise from the fact that many people have the same names. How do we know information found on Google about a patient is actually about them? If you Google one of our names (G.A.L.), an online namesake is a character from the film ‘The Devil's Advocate’, in fact ‘Alice Lomax’ in the film is Satan's child's mother. We doubt anyone is actually confused by this, but clearly less obvious confusions might happen, and the simple answer is that identities online cannot be absolutely confirmed. Furthermore an individual may use pseudonyms, or internet information might obviously be wrong, as anyone can post anything.

If, however, we had asked for informed consent from the patient to do the search in the first place, especially if they were present during the search, the potential for confusion could be reduced because they could identify any obvious errors with ease. The patient could also more openly discuss with their doctor any negative – and potentially defamatory – comments posted about them online by other people and it would also help doctors identify situations where the patient was the victim of ‘cyber-bullying’.

## Delusions of grandeur or reality?

An internet search can act as a form of collateral history. For example consider a man who presents with an exuberant, energetic persona, speaks rather quickly and loudly, and then tells his doctor he knows some Royals and has written a famous book or been in a film. Googling his name might immediately clarify whether these were grandiose delusions and this information might also make a difference in determining whether or not the patient is diagnosed with mania in the context of bipolar disorder.

Clearly the difficulty with this is that something being online does not mean it is true. It is possible to ‘be who you want to be’ online; to invent an ideal persona or avatar is almost as simple as revealing information about yourself that ‘you did not want to be made public’. However, we suspect that many clinical psychiatrists have used Google for this purpose before, as often multiple references, or references on trusted sites, can give reassurance that what someone is saying is true. There is a clear negative side to this however. Patients in psychiatry may be particularly vulnerable to not being ‘believed’ and routine Googling to check what the person has said might reinforce this tendency and stigma.

## Mental health monitoring using social media

Consider a long-term patient with severe depression, who has regular appointments with a community psychiatry team. Could someone from the mental health team monitor the patient's mental state via their social media feed or blog, with their consent? Assuming people write honestly and use the same websites regularly, social media can give a unique, time-relevant insight into a person's mental state. For example a Facebook ‘status’ or a ‘tweet’ on Twitter might often include information about how a person is feeling. The posting of certain pictures and videos or even ‘emoticons’ (cartoon faces depicting different emotions) might also reveal important insights into the patient's current frame of mind.

Clearly, if psychiatrists were to monitor mental state in this way, it would fundamentally change how mental health systems work, but it is not as far fetched as it sounds. It would not necessarily involve a person constantly watching the millions of messages streaming via a forum, Facebook or Twitter feed, which would clearly be impossible. The technology to automatically flag the use of certain phrases in emails or on social media already exists, and a team at Dartmouth University in the USA, involving computer scientists and psychiatrists are developing this technology to help prevent suicide, as part of *The Durkheim Project*.^24^

Familiarity with the internet does depend on age. Marc Prenksy describes ‘digital natives’ as compared with ‘digital immigrants’, born before the ‘rapid dissemination of digital technology in the last decades of the 20th Century’.^25^ He, fairly terrifyingly asserts that today's average university graduate has ‘spent less than 5,000 hours of their lives reading, but over 10,000 hours playing video games [and] 20,000 hours watching TV’ and that ‘as a result of this ubiquitous environment and the sheer volume of their interaction with it … think and process information fundamentally differently from their predecessors’. There is evidence that young people who self-harm find it easier to express their feelings honestly and openly in an online forum than during a face-to-face consultation and would prefer this.^26^

This suggests to us that we should be open to different methods of communication with different age groups, as not doing so means we may miss vital information. In the mastectomy case described above, George, in the paper with Volpe and colleagues, goes further, and suggests we should use all the resources we have where there are ‘red flags’, and that not using an internet search would be negligent in some cases.^21^ This tracking would, potentially, allow interventions to be made, for example to intervene urgently if a patient was suicidal. Clearly the difficulty with this is that doctors cannot check the online ‘statuses’ of all their patients all the time, and it would be difficult to gauge where responsibilities would stop and what the standard duty of care amount to in such cases. In addition, tracking a patient's blog, or social media feed might actually, quite rightly, increase a sense of paranoia.

## Safeguarding vulnerable adults online

Given that anyone and everyone can read what is openly online, an online search can sometimes protect vulnerable adults from abuse from others. Cyber-bullying, for example, involves threatening or derogatory messages posted on social media sites or online chat forums. It might also include things like encouragement to lose weight in anorexia nervosa or messages inciting self-harm or violence. Discussing this issue openly with patients and carers, in the same way that psychiatrists would openly discuss other risk issues, seems sensible. Also imagine a young man with paranoid schizophrenia who is in hospital, very unwell with psychosis, and finds it frustrating that no one ‘believes’ what he is experiencing. He tells his psychiatrist to look at his blog online, in order to full understand what he means. The team agree that with informed consent it is acceptable to do this search and they proceed with the online search. In the process they find that the blog, which has almost daily entries, gives good information about when the patient started to become unwell since there is marked evidence that his thought disorder and delusional beliefs increased in severity over the course of the past few weeks. However, the team also see that in one blog post, the patient has included sensitive personal information about himself, including his home address. They discuss this with him, and how vulnerable this might potentially make him, and arrangements are made for the blog post to be taken down.

In this situation the patient has given permission for the online search and has, in fact, asked the team to specifically read his online blog. In such cases searching online for information seems reasonable. However, this kind of scenario raises deeper ethical questions about whether mental health professionals should be proactively discussing online presence with patients, not just to find out information about deteriorating mental state, or to help holistically with recovery, but also to safeguard vulnerable individuals and potentially assess risk to others, for example by discovering threats made online in the context of illness.

## Only Googling when there is no other option

Searching for information about a patient online should also clearly be done on a ‘need to know’ basis and not purely out of curiosity or voyeurism. Imagine a core trainee being called to a forensic psychiatry in-patient unit on-call, which they do not usually work on, to examine a patient who might have a chest infection. We would not expect this doctor to search online to find out what crime was committed by the patient because this information has no bearing on the patient's physical problem and will not help the doctor to provide whatever treatment the patient may need. In fact, the internet search might even have an impact on the ability of the doctor to treat the patient in an impartial and non-judgemental manner, especially if the crime was especially heinous.

Of course, doctors have the right to protect themselves from harm and the need to perform a risk assessment may mean that the doctor would need to know about any danger posed by the patient. However, there would be no indication for an internet search in this case as other members of the team would know the patient's history well and would be able to inform the doctor if the patient was dangerous. The doctor could also, of course, consult the patient's notes if no other team members were available to consult.

## To consent or not to consent?

Would it be better practice to routinely ask consent and is informed consent possible for a Google search in psychiatry? The key elements of consent for an intervention in medicine usually include patient competence, the health professional giving clear information about potential benefits and risks and voluntariness. Many psychiatric patients fulfil all of these requirements and could, thus, consent to an online search. However, some of the patients in whom an online search may be a useful ‘investigation’ may not have the mental capacity to consent.

Likewise, patients on a psychiatry ward or in clinic may feel coerced into allowing an internet search, feeling that a ‘no’ will mean doctors will get suspicious or carry out a more ‘invasive’ online search without their consent. It is also worth pointing out that some patients might want to delete a few posts and images before the search is carried out because they deem some information to be ‘embarrassing’ (for example a photograph of them when they were an ‘awkward’ teenager). This does not seem unreasonable but it might be difficult if the doctor wants to conduct the search immediately. We are also concerned about the discussion about risks and benefits: if we do not know exactly what we will look for or find, is discussing the relative merits of a search possible? It is unusual for a doctor to discuss every possible finding of a magnetic resonance imaging scan or blood test with a patient before carrying it out, but of course the standard expected would be that relevant information is shared.

Informing patients would neuter the problem associated with deceit, however, it would not deal with the problem of potential boundary violations and it would not solve the potential for coercion either. The only way around these problems would be to seek consent from every single patient and make it clear that any refusal would be honoured. In other words, perhaps we should seek consent to search online for information about patients just like we ask for consent to speak to a relative or friend to discuss a patient's condition? If we were to do this openly, perhaps the risk that patients might become upset or angry about the process might be reduced and, as Chretien & Kind note, this would help to limit foreseen harms.^27^ There may be situations where risk to the patient, or to others, means that a Google search is appropriate without the patient's consent.

## Mental health specific concerns

Some of the ethical issues raised may be more pertinent to psychiatry than to other branches of medicine. Many patients will have experienced the validity of what they are saying being doubted by their doctors. If psychiatrists embrace PTG it could be seen as another paternalistic intervention. The capacity of patients in psychiatry will by the nature of their conditions be more likely to be impaired than in other branches of medicine. They may well lack capacity to understand the consequences of what they post on Facebook if, for example, manic and may cause damage to work and social relationships as a result. This raises difficult questions for concerned family and professionals about looking at posts on the internet, and even trying to get posted information removed.

## Conclusions and proposals for future research

Many questions remain unanswered about the acceptability of Googling patients, especially those with mental ill-health, from an ethical and legal point of view. There is clearly an urgent need for this topic to be addressed in the ethics and medical law literature.

Should psychiatrists Google patients at all? Should they do it routinely, for all their patients? Should they ask for consent each and every time? Should they share the information with the patient? These kinds of questions urgently need to be addressed by ethicists and psychiatrists alike. We feel that when making a decision to Google a patient, it would be appropriate to work through a list of questions and reflect on how one would respond, the most important question being ‘why am I doing this internet search, and is it likely to help my patient?’

Further analysis of the legality of conducting Google searches is also needed. Given that the kind of online searches we are talking about here would only involve looking for information that is already in the public domain it is not clear that this activity could be considered unlawful. However, the lack of case law in this area makes the legality of the activity harder to judge.

We feel that clear guidelines are needed from the bodies that regulate health professionals on the use of internet searching, and where these newly emerging doctor-patient boundaries lie, especially within psychiatry. We propose that more empirical research is needed on this topic. For example, we would like to know how widespread the practice of PTG is among health professionals, and whether this varies depending on age, experience or professional group. Much more qualitative information is needed about the views of patients, their friends and families, and healthcare professionals about this kind of activity: the problems it might cause and potential benefits.

Failing to make use of modern technology when this technology can improve patient care is not an option. Failing to discuss the merits and demerits of using online searchers in an open and honest fashion is not really an option either. The reality is that the internet has become an integral part of our daily lives and medicine as a whole, and psychiatry in particular, need to get to grips with what this means for modern medical practice.

## References

[1] Sanou B The World in 2014: ICT Facts and Figures. Internal Telecommunication Union, 2014 (http://www.itu.int/en/ITU-D/Statistics/Documents/facts/ICTFactsFigures2014-e.pdf).

[2] Facebook Facebook Newsroom Company Information. Facebook, 2014 (http://newsroom.fb.com/company-info/).

[3] Matthews D Internet Access – Households and Individuals, Social networking: The UK as a Leader in Europe. Office for National Statistics, 2013 (http://www.ons.gov.uk/ons/rel/rdit2/internet-access-households-and-individuals/social-networking-the-uk-as-a-leader-in-europe/sty-social-networking-2012.html).

[4] Santana S Lausen B Bujnowksa-Fedak M Rasmussen J Online communication between doctors and patients in europe: status and perspectives. J Med Internet Res 2010; 12: e20. 20551011 10.2196/jmir.1281 PMC2956231

[5] Brookes RG Menachemi N Physicians' use of email with patients: factors influencing electronic communication and adherence to best practices. J Med Internet Res 2006; 8: e2. 16585026 10.2196/jmir.8.1.e2 PMC1550692

[6] Thorp SR Fiddler J Moreno L Floto E Agha Z Lessons learned from studies of psychotherapy for posttraumatic stress disorder via video teleconferencing. Psychol Serv 2012; 9: 197-9. 22662733 10.1037/a0027057

[7] Greysen SR Chretien KC Kind T Young A Gross CP Physician violations of online professionalism and disciplinary actions: a national survey of state medical boards. JAMA 2012; 307: 1141-2. 22436951 10.1001/jama.2012.330

[8] Moubarak G Guiot A Benhamou Y Benhamou A Hariri S Facebook activity of residents and fellows and its impact on the doctor-patient relationship. J Med Ethics 2011; 37: 101-4. 21160080 10.1136/jme.2010.036293

[9] General Medical Council Doctors Use of Social Media. GMC, 2013 www.gmc-uk.org/Doctors_use_of_social_media.pdf_51448306.pdf).

[10] British Medical Association Using Social Media: Practical and Ethical Guidance for Doctors and Medical Students. BMA, 2013 (http://bma.org.uk/practical-support-at-work/ethics/ethics-a-to-z).

[11] Canadian Medical Association Social Media and Canadian Physicians – Issues and Rules of Engagement. CMA, 2013 (http://www.cma.ca/socialmedia).

[12] American Medical Association Professionalism in the Use of Social Media. AMA, 2012 (http://www.ama-assn.org//ama/pub/physician-resources/medical-ethics/code-medical-ethics/opinion9124.page).

[13] Australian Medical Association Social Media and the Medical Profession: A guide to online professionalism for medical practitioners and medical students. A Joint Initiative of the Australian Medical Association Council of Doctors-in-Training, the New Zealand Medical Association Doctors-in-Training Council, the New Zealand Medical Students' Association and the Australian Medical Students' Association. Australian Medical Association, 2010 (https://ama.com.au/social-media-and-medical-profession).

[14] Carlson N At Last – The full story of how facebook was founded. Business Insider 2010; 5 3 (www.businessinsider.com/how-facebook-was-founded-2010-3Awe-can-talk-about-that-after-i-get-all-the-basic-functionality-up-tomorrow-night-1).

[15] Wairrach HJ When doctors ‘google’ their patients. NY Times 2014; 6 1 (http://well.blogs.nytimes.com/2014/01/06/when-doctors-google-their-patients-2/?_php=true&_type=blogs&_r=0).

[16] Adams K Yes, I sometimes Google my patients. Is this surprising? Guardian 2014; 8 1 2014 (http://www.theguardian.com/commentisfree/2014/jan/08/google-patients-gp-rapport-pitfalls).

[17] Ho J Should doctors ever Google their patients? KevinMD.com 2014; 16 1 (http://www.kevinmd.com/blog/2014/01/doctors-google-patients-2.html).

[18] Mostaghimi A Crotty BH Landono BE The availability and nature of physician information on the internet. J Gen Intern Med 2010; 25: 1152-6. 20544300 10.1007/s11606-010-1425-7 PMC2947633

[19] Tang H Ng JHK Googling for a diagnosis—use of Google as a diagnostic aid: internet based study. BMJ 2006; 333: 1143. 17098763 10.1136/bmj.39003.640567.AE PMC1676146

[20] Clinton BK Silverman BC Brendel DH Patient-targeted googling: the ethics of searching online for patient information. Harv Rev Psychiatry 2010; 18: 103-12. 20235775 10.3109/10673221003683861

[21] Volpe R Blackall G Green M George D Baker M Kauffman G Googling a patient. Hastings Cent Rep 2013; 43: 14-5 (http://onlinelibrary.wiley.com/doi/10.1002/hast.206/full). 24092585 10.1002/hast.206

[22] Farnan JM Snyder Sulmasy L Worster BK Chaudhry HJ Rhyne JA Arora VM Online medical professionalism: patient and public relationships: policy statement from the American College of Physicians and the Federation of State Medical Boards. Ann Intern Med 2013; 158: 620-7. 23579867 10.7326/0003-4819-158-8-201304160-00100

[23] Gabbard GO Kassaw KA Perez-Garcia G Professional boundaries in the era of the internet. Acad Psychiatry 2011; 35: 168-74. 21602438 10.1176/appi.ap.35.3.168

[24] Poulin C Shiner B Thomson P Vepstas L Young-Xu Y Goertzel B Predicting the risk of suicide by analyzing the text of clinical notes. PLoS One 2014; 9: e85733 24489669 10.1371/journal.pone.0085733 PMC3904866

[25] Prensky M Digital natives, digital immigrants. On the Horizon 2001; 9: 1-6.

[26] Jones R Sharkey S Ford T Emmens T Hewis E Smithson J Online discussion forums for young people who self-harm: user views. Psychiatrist 2011; 35: 364-8.

[27] Chretien KC Kind T Social media and clinical care: ethical, professional, and social implications. Circulation 2013; 127: 1413-21. 23547180 10.1161/CIRCULATIONAHA.112.128017

